# Decellularised plant scaffolds facilitate porcine skeletal muscle tissue engineering for cultivated meat biomanufacturing

**DOI:** 10.1038/s41538-024-00262-1

**Published:** 2024-05-03

**Authors:** Priyatharshini Murugan, Wee Swan Yap, Hariharan Ezhilarasu, Ratima Suntornnond, Quang Bach Le, Satnam Singh, Jasmine Si Han Seah, Pei Leng Tan, Weibiao Zhou, Lay Poh Tan, Deepak Choudhury

**Affiliations:** 1 Biomanufacturing Technology, Bioprocessing Technology Institute (BTI), Agency for Science, Technology and Research (A*STAR), 20 Biopolis Way, 138668 Singapore, Singapore; 2 School of Materials Science and Engineering, Nanyang Technological University, Singapore, Singapore; 3 Department of Food Science and Technology, National University of Singapore, Singapore, Singapore

**Keywords:** Tissues, Biomaterials - cells

## Abstract

Cultivated meat (CM) offers a sustainable and ethical alternative to conventional animal agriculture, involving cell maturation in a controlled environment. To emulate the structural complexity of traditional meat, the development of animal-free and edible scaffolds is crucial, providing vital physical and biological support during tissue development. The aligned vascular bundles of the decellularised asparagus scaffold were selected to facilitate the attachment and alignment of murine myoblasts (C2C12) and porcine adipose-derived mesenchymal stem cells (pADMSCs). Muscle differentiation was assessed through immunofluorescence staining with muscle markers, including Myosin heavy chain (MHC), Myogenin (MYOG), and Desmin. The metabolic activity of Creatine Kinase in C2C12 differentiated cells significantly increased compared to proliferated cells. Quantitative PCR analysis revealed a significant increase in Myosin Heavy Polypeptide 1 (MYH1) and MYOG expression compared to Day 0. These results highlight the application of decellularised plant scaffold (DPS) as a promising, edible material conducive to cell attachment, proliferation, and differentiation into muscle tissue. To create a CM prototype with biological mimicry, pADMSC-derived muscle and fat cells were also co-cultured on the same scaffold. The co-culture was confirmed through immunofluorescence staining of muscle markers and LipidTOX staining, revealing distinct muscle fibres and adipocytes containing lipid droplets respectively. Texture profile analysis conducted on uncooked CM prototypes and pork loin showed no significant differences in textural values. However, the pan-fried CM prototype differed significantly in hardness and chewiness compared to pork loin. Understanding the scaffolds’ textural profile enhances our insight into the potential sensory attributes of CM products. DPS shows potential for advancing CM biomanufacturing.

## Introduction

Cultivated meat (CM), also known as cultured meat, clean meat, or cell-based meat, is an innovative technology revolutionising meat production through cell cultures^1–4^. It addresses sustainability concerns in traditional animal-based meat production methods, tackling issues such as antibiotic overuse, food and water security, safety, environmental impact, and animal welfare^4,5^. Tissue engineering techniques play a crucial role in replicating muscle development in a laboratory setting, necessitating the seeding of cells within an environment that mimics the natural conditions found in native tissues^6^. This approach is critical in successfully producing CM, enabling the growth of meat tissues outside a living animal and potentially leading to significant advancements in meat innovation. Recently, the United States Department of Agriculture approved CM production and sale, allowing two California-based companies to offer chicken products made from chicken cells^7,8^. This decision positions the US as the second country, following Singapore, to authorise CM production and sale^9,10^.

Structured CM product scalability relies on four key elements: cells, medium, scaffold, and bioprocessing^4^. The scaffold is critical for scaling up CM production by providing a platform for cultured cells to grow. Macro-porous scaffolds support cellular proliferation and offer essential mechanical support^4,11–14^. There is therefore a need to develop unique macro-porous scaffolds that may resemble meat in terms of texture, flavour, and nutritional value^15^. Techniques like anisotropic architecture, hybrid multi-materials, and surface patterning can enhance scaffold texture, promoting cell alignment and myotube formation^6^. Through mimicking natural tissue architecture, scaffolds facilitate product development by improving texture and overall meat-like characteristics, meeting consumers’ demands for realistic and appealing CM options^14^.

The ideal scaffold composition should consist of edible or biodegradable materials that can break down into molecules with favourable organoleptic profiles. To align with the sustainability goals of CM, plant-based ingredients present a promising solution. Decellularisation of plant tissue removes cellular components while preserving the overall structure of the tissues, resulting in a scaffold with inherent structural complexity. These sustainable scaffolds, sourced from plants, reduce reliance on animal agriculture, thereby minimising environmental impacts. Their abundance and accessibility make them a viable resource for large-scale CM production^16–18^. Previous studies have explored the potential of decellularised spinach leaves and grass as edible scaffolds for culturing primary bovine satellite cells and the murine myoblast cell line C2C12. These scaffolds provided a vascular network that supported cell growth. However, their limited thickness poses a challenge when attempting to construct substantial meat samples, requiring the stacking of multiple layers^19,20^. Furthermore, decellularised broccoli florets have been used as microcarriers for culturing primary bovine satellite cells, exhibiting successful cell adhesion and viability in dynamic cultures. However, the findings from this research did not demonstrate the potential of the decellularised floret in promoting muscle differentiation^21^.

While current CM products mainly resemble minced, powdered, or scaled-down versions of natural meat cuts, there is a real demand for natural, edible decellularised plant scaffolds (DPS) that are cost-effective and suitable for large-scale production, particularly to mimic voluminous and structured conventional meat^22–24^. This study thus addresses the existing research gap by developing a CM prototype comprising of decellularised plant-based scaffold populated with co-cultured muscle and fat cells. A decision matrix was created to assist in selecting suitable plant and fungi materials for decellularisation based on factors like edibility, digestibility, availability, pore density, rigidity, cell alignment features and viable cell growth. These criteria ensure the selection of stable scaffolds capable of supporting cell growth and allowing nutrients and gaseous exchange. Plant and fungi scaffolds, including asparagus and mushrooms, were developed. Asparagus was chosen as a proof-of-concept (POC) for its vascular bundle’s arrangement, providing cell alignment, rigidity, and resilience during cooking. The DPS with a macro-connected porous structure was carefully modified to enhance the growth and development of skeletal muscle and fat cells, aiming to create a CM prototype resembling traditional meat.

Thorough evaluations were conducted to assess the efficacy of these DPS, including biocompatibility assessments, determination of muscle differentiation potential through immunofluorescence staining, analysis of gene expression related to muscle development, and quantification of muscle protein production. Scaffolds’ durability during cooking processes and textural properties were assessed for food application suitability. The success of this study marks a significant milestone in the development of decellularised plant-based hybrid CM prototypes that could potentially be scaled up. In future, the DPS could be easily produced from various edible species of plants in different shapes and dimensions and hence could be adapted for various bioreactor configurations for scale-up CM manufacturing.

## Results

### DPS characterisation

**Fig. 1:**
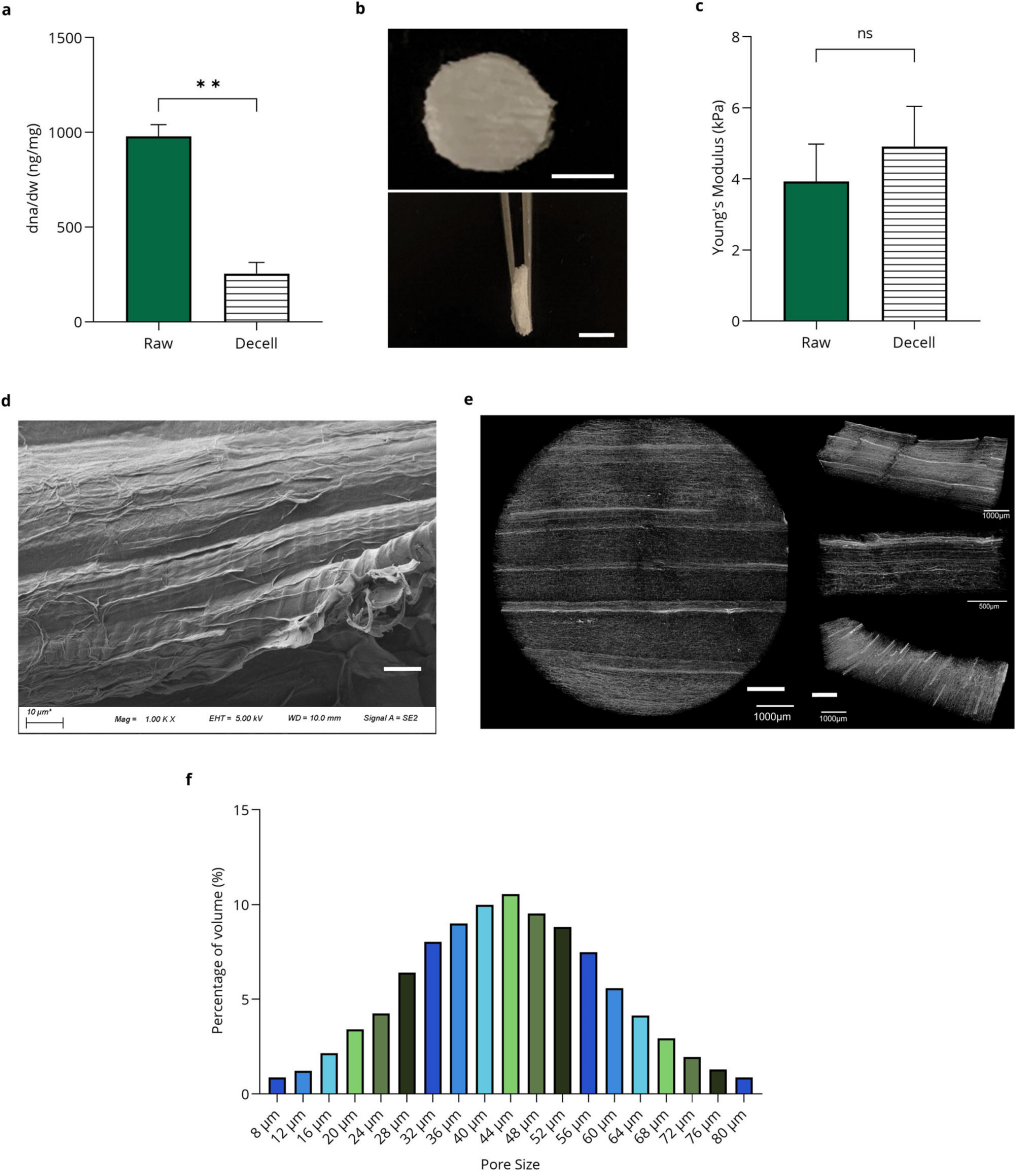
DPS characterisation. **a** DNA quantification before and after decellularisation; Data are presented as means ± standard deviation (s.d.) (*n* = 2; **P < 0.01) and were analysed using Welch’s test. **b** Visual image of DPS ( ≈ 10 mm diameter and ≈2 mm thickness) scale bar = 5 mm, top–top view, bottom–side view. **c** Young’s modulus testing at a 10% strain rate and 10% compression of raw and DPS; Data are presented as means ± standard deviation (s.d.) (*n* = 5; ns, not significant) and were analysed using one-way ANOVA followed by Brown–Forsythe and Bartlett’s test**. d** SEM images of the scaffold, scale bar = 10 µm**. e** Micro-CT 3D scan of the DPS, left—top view, and right—side view (top, middle, and bottom layers, respectively), scale bar = 1000 µm. **f** Percentage of pore volume area as a function of pore diameter based on Micro-CT analysis.

DNA quantification of the DPS showed that decellularisation significantly removed plant DNA from the native asparagus tissue (Fig. 1a). Raw (non-decellularised) samples had an average DNA content of 978 ± 62 ng/mg. In comparison, DPS retained an average DNA content of 254 ± 60 ng/mg. Data are presented as means ± standard deviation (s.d.) (*n* = 2; **P < 0.01) and were analysed using Welch’s test. Based on Fourier transform infrared (FTIR) results, scaffolds primarily consisted of cellulose, hemicellulose, and pectin (Supplementary Fig. 1). The percentage of carbon, oxygen, and other elements from energy dispersive X-ray (EDX) analysis in both raw and DPS was discussed (Supplementary Fig. 2). After freeze-drying the DPS (*n* = 5), they exhibit an average diameter of 10.1 ± 0.6 mm and a thickness of 2.0 ± 0.5 mm (Fig. 1b) and the visual image of 100 scaffolds is shown in Supplementary Fig. 3. The DPS has Young’s modulus of 4.9 ± 1.12 kPa with *P*-value of 0.1747 (Fig. 1c) which lies between the ideal Young’s modulus conditions required for adipogenic differentiation (around 3 kPa) and for myoblast growth and differentiation (10 kPa for proliferation and 18 kPa for differentiation)^12,25^. Data are presented as means ± standard deviation (s.d.) (*n* = 5; ns, not significant) and were analysed using one-way ANOVA followed by Brown–Forsythe and Bartlett’s test. Scanning electron microscopy (SEM) image shows the surface topography of the DPS (Fig. 1d). Upon longitudinal cutting of the asparagus stem, an increase in the visibility of vascular bundles was observed after decellularisation, resulting in scaffolds with horizontally aligned vascular bundles that facilitate favourable cell attachment and alignment. Micro-computed tomography (Micro-CT) images show the alternating structure of vascular bundles and ground tissue present throughout the 3D scaffold, with vascular bundles running along the same direction (Fig. 1e). Since asparagus is a monocot that is characterised by scattered vascular bundles around parenchyma, the observed arrangement is expected^26^. Analysis of the DPS pore size distribution (Fig. 1f) revealed that the pores ranged between 8 and 80 μm in diameter, with the highest proportion of pores being 44 μm which accounts for 10.5% of the total scaffold volume. 3D quantification of the Micro-CT scans in Table 1 shows that the DPS is highly porous with ~93.5% porosity compared to the total volume. The pores in the DPS were highly connected, with connectivity values reaching 93.55%.

**Table 1:** Micro-CT analysis of scaffold for different parameters

Feature	Value
Number of closed pores	602
Volume of closed pores	17,065 µm^3^
Closed porosity	0.0015%
Volume of open pore space	16,438,000,000 µm^3^
Percentage of open porosity/connectivity	93.55
Percentage of open surface area	99.99
Total volume of open pore space	16,438,000,000 µm^3^
Percentage total porosity	93.5

### Assessment of C2C12 myoblast proliferation and differentiation on DPS

**Fig. 2:**
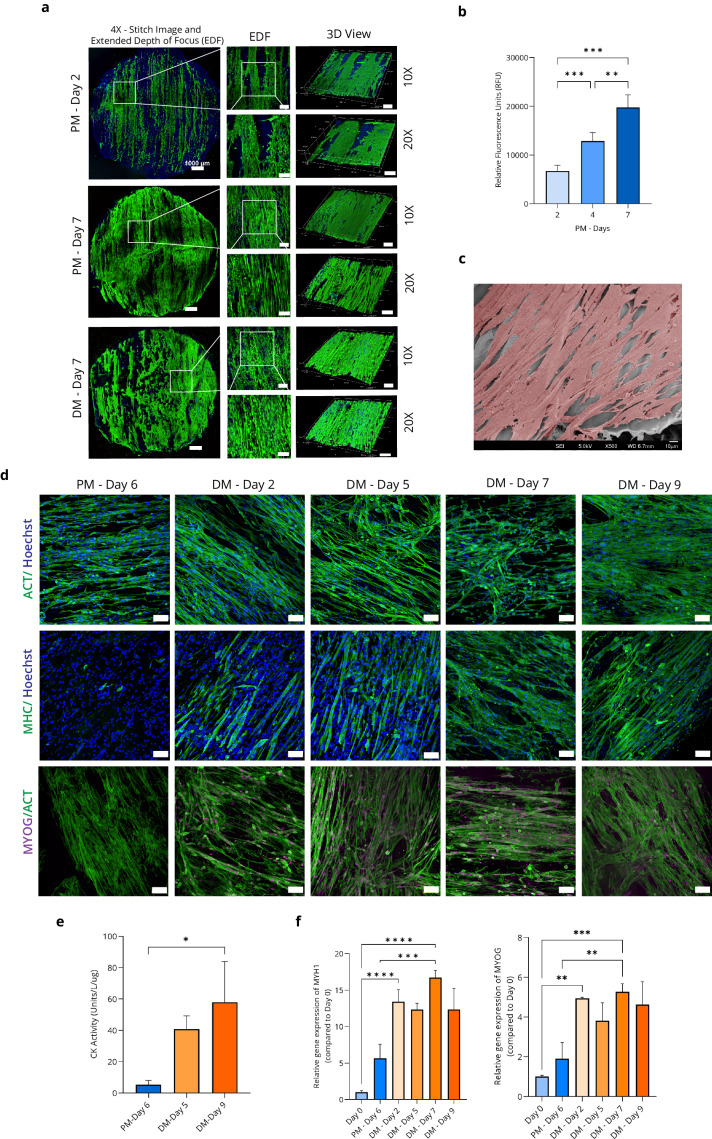
Assessment of C2C12 myoblast proliferation and differentiation on DPS. **a** Confocal images show the live/dead/nuclei staining of C2C12 cells cultured on the DPS using proliferation media (PM) and differentiation media (DM) (*n* = 3), live cells (green), dead cells (red), and nucleus (blue), scale bar at various magnifications: ×4—1000 µm, ×10—250 µm, ×20—150 µm. **b** Cell viability (*n* = 5); Data are presented as means ± standard deviation (s.d.) (*n* = 5; **P < 0.01; ***P < 0.001) and were analysed by Brown–Forsythe and Welch ANOVA test. **c** SEM image of C2C12 differentiated muscle cells in DM—Day 9 (muscle cells were pseudo-coloured in red), scale bar = 10 µm. **d** Representative immunofluorescence images of C2C12 cells cultured on the DPS using PM and DM. Actin, ACT (green); Myosin Heavy Chain, MHC (green); Myogenin, MYOG (far-red); Hoechst (blue). Scale bar = 100 µm (×20 magnification) (*n* = 3). **e** Creatine kinase (CK) activity; Data are presented as means ± standard deviation (s.d.) (*n* = 3; *P < 0.05) and were analysed using one-way ANOVA followed by Tukey’s multiple comparisons test. **f** qPCR gene expression analysis of Myosin Heavy Polypeptide 1 (MYH1) (left) and MYOG (right) (*n* = 3); Data are presented as means ± standard deviation (s.d.) (*n* = 3; **P < 0.01, ***P < 0.001, ****P < 0.0001) and were analysed using one-way ANOVA followed by Tukey’s multiple comparisons tests.

The proliferation and differentiation of C2C12 myoblasts in 3D culture using DPS were evaluated. Live/dead imaging was performed on days 2 and 7 in the proliferation medium (PM) and on day 7 of the differentiation media (DM) to assess cell viability and attachment. Confocal microscope images demonstrated strong cell attachment across the scaffold, with cells aligning along the direction of the vascular bundle (Fig. 2a and Supplementary Fig. 4). By Day 7 of PM, the cells had covered both sides of the scaffolds and achieved confluency. The PrestoBlue^TM^ assay confirmed the biocompatibility of the DPS. Cell viability increased 2.94-fold from Day 2 to Day 7, indicating its ability to support cell growth and metabolic activity (Fig. 2b). Data are presented as means ± standard deviation (s.d.) (*n* = 5; **P < 0.01; ***P < 0.001) and were analysed by Brown–Forsythe and Welch ANOVA test. SEM imaging demonstrates that the scaffold with differentiated C2C12 cells exhibited prominent myotubes aligned in a regular pattern along the scaffolds. To visualise the C2C12 cells distinctly on the scaffold, the cells were pseudo-coloured in red (Fig. 2c, raw data-Supplementary Fig. 9). The immunocytochemistry analysis of differentiated C2C12 cells on scaffolds revealed significant protein expressions specific to late stages of muscle differentiation, such as myosin heavy chain (MHC) and myogenin (MYOG). In the proliferation medium (PM—Day 6), no expression of MHC and MYOG was observed. However, upon switching to a differentiation medium (DM), the markers began to express on DM-Day 2, with a stronger signal on DM—Day 5, 7, and 9 with successful visualisation of multi-nucleated myotube formation (Fig. 2d).

Creatine kinase (CK) is an enzyme specific to muscle and is generally used as an indicator of muscle cell differentiation^27^. To evaluate the effectiveness of the scaffold in promoting muscle cell differentiation, the metabolic activity of CK was measured in C2C12 cells cultured on the scaffold. CK activity consistently increased throughout the differentiation period, reaching its peak on DM—Day 9, showing a remarkable 10.92-fold increase compared to PM—Day 6 (Fig. 2e). Data are presented as means ± standard deviation (s.d.) (*n* = 3; *P < 0.05) and were analysed using one-way ANOVA followed by Tukey’s multiple comparisons tests. This observation confirms the successful myogenic differentiation of the C2C12 cells into muscle cells. Quantitative PCR (qPCR) analysis of muscle-related genes, including myosin heavy polypeptide 1 (MYH1) and MYOG, demonstrated a significant increase in their expression in differentiated C2C12 muscle cells on scaffolds compared to proliferating C2C12 cells (Fig. 2f). Specifically, on DM—Day 7, MYH1 expression increased by 16.7-fold compared to Day 0 and 2.98-fold compared to PM-Day 6. Moreover, MYOG expression increased by 5.3-fold compared to Day 0 and 2.78-fold compared to PM—Day 6. Data are presented as means ± standard deviation (s.d.) (*n* = 3; **P < 0.01, ***P < 0.001, ****P < 0.0001) and were analysed using one-way ANOVA followed by Tukey’s multiple comparisons tests.

### Porcine adipose-derived mesenchymal stem cells (pADMSCs) myogenesis on DPS (CM prototype)

The proliferation and differentiation protocol of porcine adipose-derived mesenchymal stem cells (pADMSCs) into muscle cells in a 3D culture using DPS was evaluated. pADMSCs were isolated^28^ (Supplementary Fig. 5a). Their multipotency for differentiation into adipocytes, osteocytes, and chondrocytes was characterised using oil-red staining, alizarin red staining, and toluidine blue staining respectively^28^ (Supplementary Fig. 5b). The two-dimensional differentiation of pADMSCs into muscle cells was evaluated using immunofluorescence staining and shown in Supplementary Fig. 6, employing muscle-specific antibodies (MHC, DES, MYOG).

**Fig. 3:**
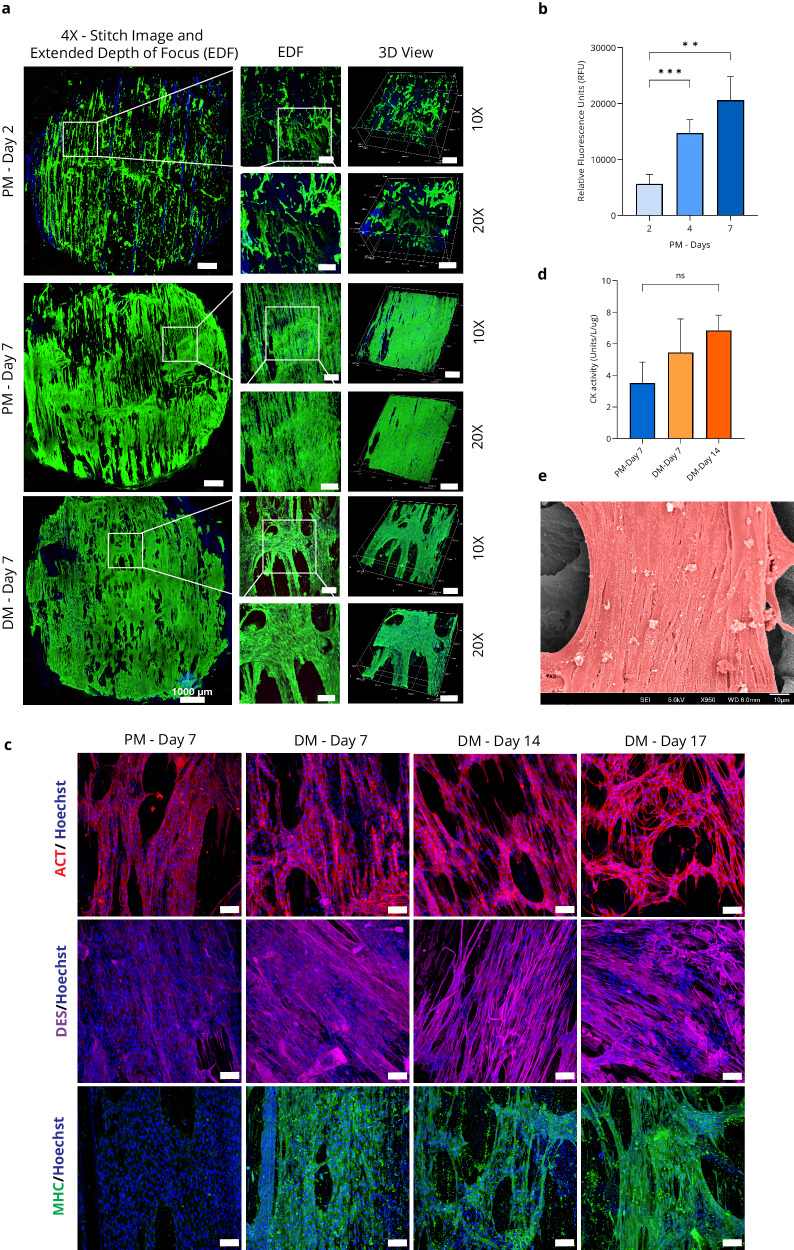
Porcine adipose-derived mesenchymal stem cells (pADMSCs) myogenesis on DPS (CM prototype). **a** Confocal images show the live/dead/nuclei staining of pADMSC cells cultured on the DPS using proliferation media (PM) and differentiation media (DM) (*n* = 3), live cells (green), dead cells (red), and nucleus (blue), scale bar at various magnifications: ×4—1000 µm, ×10—250 µm, ×20—150 µm. **b** Cell viability (*n* = 5); Data are presented as means ± standard deviation (s.d.) (*n* = 5; **P < 0.01; ***P < 0.001) and were analysed by Brown–Forsythe and Welch ANOVA test. **c** Representative immunofluorescence images of pADMSC cells cultured on the DPS using PM and DM. Actin, ACT (red); Myosin Heavy Chain, MHC (green); Desmin, DES (far-red); Hoechst (blue). Scale bar, 100 µm (×20 magnification) (*n* = 3). **d** Creatine kinase (CK) activity was assessed throughout the cultivation period to evaluate myogenic differentiation (*n* = 3), Data are presented as means ± standard deviation (s.d.) (*n* = 3; ns, not significant) and were analysed using one-way ANOVA followed by Tukey’s multiple comparisons tests. **e** SEM image of pADMSC differentiated muscle cells in DM—Day 17 on DPS where the muscle cells were pseudo-coloured in red, scale bar = 10 µm.

Live/dead/nuclei imaging was performed during proliferation on days 2 and 7 and day 7 of differentiation to assess pADMSCs cell viability and attachment. Confocal microscope images revealed robust cell attachment throughout the scaffold, with cells aligning along the vascular bundle’s direction (Fig. 3a). By Day 7 of PM, the cells had fully covered both sides of the scaffolds and reached confluency, resembling the C2C12 cell growth observed in DPS. The proliferative performance of pADMSCs in the DPS was assessed using the PrestoBlue^TM^ assay. Cell viability increased 3.64-fold from Day 2 to Day 7, confirming the DPS’s excellent biocompatibility and promoting cell growth (Fig. 3b). Data are presented as means ± standard deviation (s.d.) (*n* = 5; **P < 0.01; ***P < 0.001) and were analysed by Brown–Forsythe and Welch ANOVA test. Immunocytochemistry analysis of differentiated pADMSCs on scaffolds illustrates significant protein expression associated with muscle differentiation, including MHC and DES. The staining performed using anti-MHC and DES antibodies effectively visualised the formation of elongated myotubes with multiple nuclei (Fig. 3c). To assess the role of the DPS in promoting myogenesis, the metabolic activity of CK was measured in pADMSCs that were undergoing differentiation into muscle cells on the scaffold. Throughout the differentiation period, CK activity steadily increased, peaking on DM-Day 14 with a 1.95-fold increase compared to PM-Day 7 (Fig. 3d). Data are presented as means ± standard deviation (s.d.) (*n* = 3; ns, not significant) and were analysed using one-way ANOVA followed by Tukey’s multiple comparisons tests. Overall, these observations provide evidence of successful myogenic differentiation in the pADMSCs, confirming their transformation into muscle cells on the DPS. SEM imaging demonstrates well-defined myotubes in the scaffold with differentiated pADMSCs muscle cells aligned in the same direction as the vascular bundle of the DPS. They were pseudo-coloured in red to distinguish the muscle cells from the scaffold (Fig. 3e, raw data-Supplementary Fig. 10).

### Co-culture of pADMSCs derived muscle cells and fat cells on the DPS

**Fig. 4:**
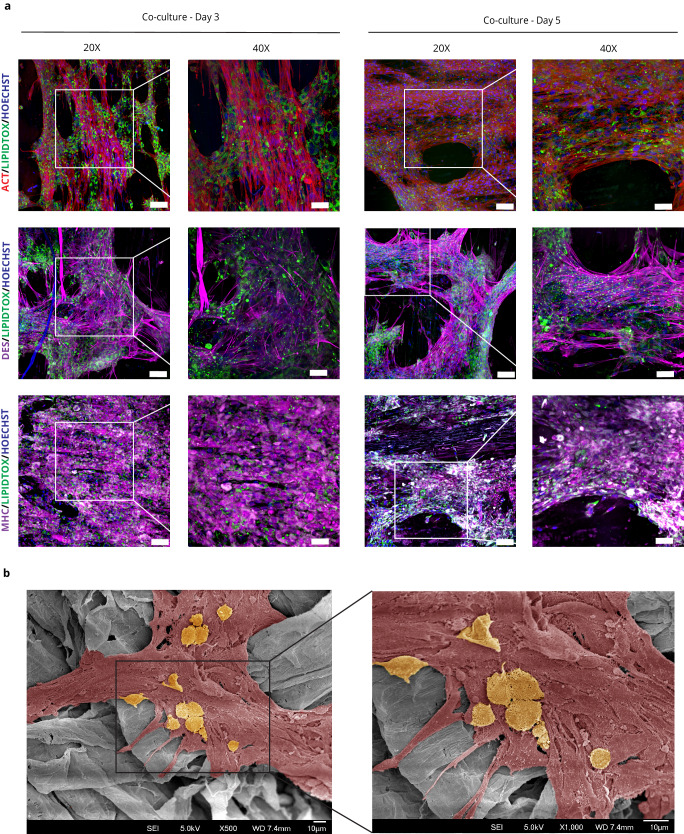
Co-culture of pADMSC-derived muscle and fat cells on the DPS. **a** LipidTOX staining of fat cells (green) with muscle markers—Desmin, DES (far-red); Myosin Heavy Chain, MHC (far-red); Actin, ACT (red) and nucleus (blue) at ×20 (scale bar = 100 µm) and ×40 magnification (scale bar = 50 µm). **b** SEM image of pADMSCs differentiated muscle cells, pseudo-coloured in red (DM—Day 22) and fat cells pseudo-coloured in yellow (scale bar = 10 µm) (co-culture—Day 5) on scaffolds, respectively.

The pADMSCs underwent differentiation into adipocytes using a similar technique developed by Zagury et al.^29^. The differentiation process was confirmed using LipidTOX Red neutral lipid stain (Supplementary Fig. 7). Subsequently, the co-culturing of pADMSC-derived muscle and adipose cells was evaluated on the DPS for 5 days. Immunofluorescence staining was performed using specific markers for muscle cells (MHC and DES), in combination with LipidTOX staining, to evaluate the co-culture (Fig. 4a). The use of LipidTOX stain allowed for the visualisation of intracellular neutral lipid accumulation, confirming the presence of adipocytes within the co-culture. In the co-culture system, pADMSC-derived adipocytes were observed to be attached on top of the muscle cells that had differentiated within the scaffold. Notably, the pADMSC-derived adipocytes and muscle cells maintained their phenotype throughout the co-culture period, as evident from observations made on Day 3 and Day 5. Additionally, SEM imaging demonstrates the attachment of round-shaped adipocytes on top of elongated muscle cells (Fig. 4b, raw data-Supplementary Fig. 11).

### Mechanical and textural evaluation of CM Prototype

**Fig. 5:**
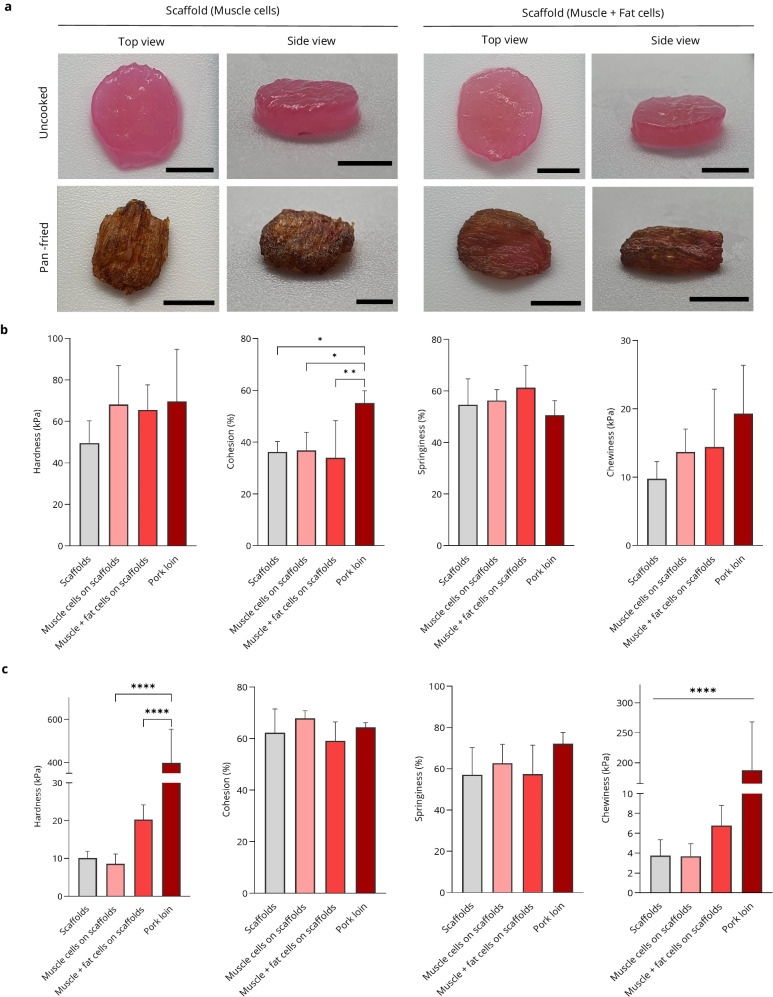
Mechanical and texture evaluation of CM prototype. **a** Representative images of uncooked and pan-fried samples containing only muscle cells or a combination of both fat cells and muscle cells (scale bar = 5 mm). Texture profile analysis (TPA) results of **b** uncooked samples and **c** pan-fried samples, Data are presented as means ± standard deviation (s.d.) (*n* = 5; *P < 0.05, **P < 0.01, ****P < 0.0001) and were analysed using one-way ANOVA followed by Tukey’s multiple comparisons tests.

Texture profile analysis (TPA) was performed on the CM prototype to gain insights into its behaviour during chewing^30^. This study also used pork loin as a benchmark against the CM prototype. To mimic the actual cooking and preparation of meat before consumption, the CM prototypes were pan-fried prior to analysis (Supplementary video 4). Thermogravimetric analysis (TGA) revealed the thermal stability of DPS, crucial for potential applications in food products, including CM subjected to high-temperature cooking conditions (Supplementary Fig. 8). Representative images of the CM prototypes before and after cooking can be found in Fig. 5a. Browning was also observed on the pan-fried CM prototypes, which could be linked to the Maillard reaction^31^ in classical meat cooking. The TPA performed on the raw meat samples (Fig. 5b) revealed no significant differences across most textural parameters (e.g., hardness, springiness, and chewiness). However, a significant difference in cohesiveness was observed between the pork loin and the CM prototypes. The TPA performed on the pan-fried CM prototypes (Fig. 5c) revealed significant differences in the hardness and chewiness between the CM prototype and the pork loin. Data are presented as means ± standard deviation (s.d.) (*n* = 5; *P < 0.05, **P < 0.01, ****P < 0.0001) and were analysed using one-way ANOVA followed by Tukey’s multiple comparisons tests.

## Discussion

Cultivated Meat, a rapidly advancing field within cellular agriculture, utilises advanced biotechnology to produce food. Using skeletal muscle tissue engineering, this transformative technology can potentially revolutionise the food industry, offering solutions to environmental and ethical challenges^1–4,20,32,33^. Decellularisation is crucial for plant scaffold preparation in CM as it removes plant cells, preserving tissue structure and ECM, enabling cell growth and scaffold functionality^34^. In the study, the DPS had an average DNA content of 254 ± 60 ng/mg exceeding the threshold of ≤50 ng/mg for thoroughly decellularised tissue proposed for regenerative medicine applications^35^. However, prior studies on decellularisation of plant scaffolds have also reported DNA content deviating from conventional standards yet demonstrated successful cell growth and proliferation^36–38^. For CM production, this criterion is non-essential, as asparagus is consumed whole, posing no known DNA danger. The decellularisation process is lengthy due to the purification phase, which aims to eliminate surfactant residue. Future work will involve the use of safer surfactants and exploring commercial decellularisation processes for streamlined processing^39,40^. DPS enhances muscle cell alignment, resulting in a structured and voluminous CM product resembling traditional cuts. This advancement aims to offer consumers a realistic and satisfying alternative to meat, meeting expectations for texture, appearance, and mouthfeel. We opted for the C2C12 mouse myoblast cell line (ATCC) as our model to assess DPS efficacy in supporting cell proliferation and muscle differentiation due to its prevalent use in muscle studies and established reliability. For the CM red meat prototype, pADMSCs were employed. The distinct muscle differentiation patterns observed in both cell types can be attributed to their inherent cellular characteristics. We developed an improved static cell seeding method for C2C12 and pADMSCs on scaffolds, achieving full coverage and maximising scaffold utilisation. After reaching confluency by Day 7, differentiation was induced with reduced serum. A cell viability assay optimised the differentiation protocol, ensuring viability while promoting muscle cell differentiation. The study concluded upon achieving both viability and complete differentiation (Supplementary Fig. 12). The integration of cell viability assessments with immunofluorescence imaging informed our conclusive timeline. The decision to exclude a 2D positive control in this study was based on the disparate cellular growth and proliferation patterns observed between the scaffold and the 2D cell culture plate (Supplementary Fig. 13).

Previous research has demonstrated using various cell types on decellularised asparagus scaffolds for human skeletal muscle tissue development. However, it employed non-edible human fibronectin for scaffold biofunctionalisation^41^. In contrast, our study adapts biofunctionalisation methods for decellularised asparagus scaffolds, including potentially edible ingredients^42^. While prior studies have shown diverse cell type’s viability on decellularised asparagus scaffolds^19,21^, it was crucial to assess the long-term survival and differentiation potential of relevant pADMSCs. Our findings confirm the feasibility of sustaining primary pADMSCs on decellularised asparagus scaffolds for extended periods with minimal cytotoxic effects. In this study, asparagus stems were cut longitudinally to create scaffolds with horizontally aligned vascular bundles, promoting cell attachment and alignment. Cells seeded within the porous stem interior are expected to adhere, proliferate, and integrate into the scaffold, facilitating myotube formation. The scaffold’s macroporous network enhances nutrition and oxygen flow, which is crucial for growing muscle tissue^41^. However, using these pores for media perfusion for scale up production is yet to be thoroughly investigated. DPS demonstrates consistent structural integrity throughout the cell culture period, providing essential mechanical support for myotube formation (Supplementary Video 1). In contrast, previous studies on textured soy protein scaffolds documented partial degradation when cultured with bovine satellite cells, resulting in increased brittleness and diminished tensile strength^43^.

To enhance meat texture and flavour, we created a co-culture system using pADMSC-derived muscle and fat cells in a single scaffold. This CM prototype brings us closer to conventional meat. Muscle cells matured first in the scaffold, followed by the introduction of adipocytes onto the same scaffold, promoting adherence and sustaining co-culture for a week. We optimized this strategy to maintain the phenotypic traits of both cell types within the scaffold (Supplementary videos 2, 3). Scaffold mechanical testing for CM was consistently performed in dry conditions to assess original properties for intended dry commercial use. However, texture profile analysis involved submerging scaffolds to mimic moisture effects like the positive control, as shown in Fig. 5. The scaffold size can be tailored, or bottom-up integration of scaffolds can be carried out to mimic standard meat, ensuring scalability in manufacturing for CM development. Our study showcased the feasibility of co-culturing muscle and fat cells together for the first time in a decellularised plant scaffold, yielding a promising CM prototype.

## Methods

### Preparation of the DPS

White asparagus (*Asparagus officinalis*) was obtained from a local grocery store. The asparagus samples were sliced using a mandolin slicer longitudinally (i.e., in the direction of its vascular bundles). A 12 mm diameter circular biopsy punch was used to create samples of consistent size. The pieces were then placed in 500 mL glass bottles and submerged in 1% w/v sodium dodecyl sulfate (SDS) (Sigma-Aldrich, USA) in deionised (DI) water for 88 h, refreshing the solution after the first 24 h. The SDS was then replaced with 1% v/v Triton-X (Biobasic, Canada) in deionised water for 24 h and 100 mM calcium chloride (CaCl_2_) (Sigma-Aldrich, USA) sequentially for another 24 h. The samples were then submerged in 70% v/v ethanol with agitation in a shaking incubator (NB-205L, N-Biotek, South Korea) for 30 min before being rinsed thrice in DI water for 15 min each. 100 mM of CaCl_2_ in DI Water was added to the samples for 24 h. The samples were then rinsed in DI water until no more visible bubbles were left behind. After collection and overnight storage at −20 °C, the decellularised samples are transferred to −80 °C in preparation for lyophilisation. Lyophilisation was performed using the FreeZone 4.5 Plus freeze-dryer (Labconco, USA) for at least 48 h. The samples were stored at −20 °C until subsequent experiments were conducted^44,45^.

### DNA quantification of DPS

Freeze-dried decellularised and raw (non-decellularised) samples were cryo-milled (Ultra Centrifugal Mill ZM 200, Retsch, Germany) to obtain a fine powder. The DNA content of the samples was measured using a CYQUANT® DNA assay kit (Thermo Fisher Scientific, Waltham, MA, USA), with slight modifications to the method described by Jones et al.^19^. To extract the DNA from the sample, the cryomilled samples were each combined with 1 mL of lysis buffer included in the CYQUANT® DNA assay kit and a disruption bead from the Dneasy® Plant Pro Kit (Qiagen, Germany) and incubated in a 37 °C water bath for 2 h. The extracted DNA was fluorescently labelled by adding 100 μL of the CYQUANT® GR dye. Decellularised samples were compared to a standard curve created by serial diluting a DNA standard and raw samples as a control. Fluorescence intensity was then measured using a fluorescence microplate reader (Infinite® 200 PRO, Tecan, Switzerland) with an excitation and emission wavelength of 480 and 520 nm, respectively. The fluorescence intensity value is then converted to DNA content using the linear regression equation obtained from the standard curve values.

### Micro-CT analysis

The scaffolds were first subjected to serial ethanol dehydration (50%, 70%, 80%, 90%, 95%, 100%) for 15 min at each concentration step. The samples were kept in 100% v/v ethanol overnight before they were dried at a critical point (Leica EM CPD 300 Automated Critical Point Dryer, Leica Microsystems, Austria). Micro-CT images of scaffolds were acquired using Skyscan 1272 (Bruker Industry, USA). The scaffolds were scanned using a source voltage of 30 kV, a source current of 150 μA, a resolution of 1.999967 μm, an exposure time of 2345 ms and a rotation step of 0.100°. Images were reconstructed using the NRecon programme (Bruker Industry, USA). The volume, surface area of the pores, and porosity were quantified and analysed using the CTAn software (Bruker Industry, USA).

### C2C12 cell culture

The C2C12 cell line, obtained from the American Type Culture Collection (ATCC CRL-1772™), was employed in this study. The cells were cultured in T75 or T175 tissue culture flasks (Corning, 430641U and 431080) and maintained in a growth medium (PM—proliferation medium) consisting of high glucose DMEM medium (Gibco, 10569010), 10% v/v foetal bovine serum (FBS) (Gibco, 16140071), and 1% v/v Penstrep (Gibco, 10378016). The incubation was conducted at 37 °C in a humidified atmosphere of 5% CO_2_. Sub-culturing of the cells occurred when they reached 50–60% confluence. For this research, cells from passages 4–6 were used.

### Isolation and expansion of pADMSCs

The protocol to isolate pADMSCs was adapted from Chen et al.^28^. Subcutaneous adipose tissues were first harvested from pigs. The tissues were finely minced and washed with Dulbecco’s phosphate-buffered saline (PBS; Gibco, Grand Island, NY, USA) before they were digested in a digestion medium containing 0.1% v/v collagenase type II (900 units of collagenase/1.5 mL DMEM/g fat) DMEM and penicillin-streptomycin (P/S; Gibco). The digestion was performed in a glass bottle, left in a shaking incubator rotating at 45 rpm at 37 °C for no more than 90 min. An equal volume of culture medium containing DMEM with 10% v/v FBS was added to stop the digestion. This slurry solution was passed through a 100 µm cell strainer into 50 mL centrifuge tubes. A pellet of stromal-vascular cells can be collected via centrifugation at 700×*g* for 10 min. The pellet was washed by adding 10 mL DMEM into each tube to resuspend the pellet with pipetting and gentle tube shaking. Collect the washed cells via centrifugation at 700×*g* for 6 min. To lyse red blood cells in the stromal-vascular fraction, 10 mL of ACK lysis buffer was added to the cell pellet and then resuspended by pipetting. After that, let it stand for 10 min at room temperature (RT). To stop the reaction, an equal amount of DMEM was added, and the tube was gently inverted twice to homogenise. The lysed cells can be collected via centrifugation at 700×*g* for 10 min. The cell pellet was rewashed with DMEM before it was collected and seeded into culture flasks at a density of 6000 cells/cm^2^ with culture medium containing DMEM supplemented with 10% v/v FBS with 200 U/mL of penicillin and 200 µg/mL of streptomycin (P/S, Gibco). Incubate the flasks in the 37 °C incubator in air with 5% CO_2_ to allow cell attachment to the plates. Fresh growth medium was replaced every 2–3 days. Upon 80% confluence, cells can be dissociated with 0.25% v/v trypsin-EDTA and sub-cultured. Porcine adipose-derived stem cells (pADMSCs) isolated were expanded in culture and may be frozen in a solution with 10% v/v dimethyl sulfoxide (DMSO; Sigma) in FBS for long-term storage. For this research, cells from passages 4–6 were used.

### Three-dimensional myogenesis of C2C12 and pADMSC cells on the DPS

The DPS were first immersed in 70% v/v ethanol for 30 min, followed by a triple wash with PBS. Subsequently, the scaffolds underwent a three-day bio-functionalisation process adapted from prior studies^42^. Finally, they were immersed in the proliferation medium (PM) for 3 days, facilitating optimal scaffold preparation and promoting robust cellular growth. The cell culture was conducted using 24-well plates with ultra-low attachment surfaces (Corning, 3473). Static cell seeding was utilised on both sides of the scaffold to establish a standardised cell seeding strategy. Initially, 100,000 C2C12 cells were seeded on the top of the scaffold, where the vascular bundles aligned parallel to the bottom well surface. This seeding process was performed with a restricted volume of media to prevent overflow and facilitate absorption by the scaffold. Following a 6 h incubation, PM (500 μL) was gradually added and allowed to remain for one day. The next day, the scaffold was flipped to seed cells on the bottom side using the same cell-seeding procedure. The cells seeded on the scaffold were cultured in PM comprising DMEM, 10% v/v FBS, and 1% v/v Penstrep for a week, with media changes every other day. Once the cells achieved ~90% confluency and completely covered the scaffolds, the media was switched to differentiation media (DM) (DMEM, 2% v/v Horse serum, and 1% v/v Penstrep)^46^. The differentiation phase was maintained for nine days, with media changes every other day. For pADMSCs, the same cell seeding strategy was followed with extended incubation of 12 h, and the cells were cultured in the PM consisting of DMEM, 20% v/v FBS, and 1% v/v Penstrep for a week, with media changes every other day. Once the cells reached ~90% confluency and completely covered the scaffolds, the media was switched to DM and maintained for 17 days, with media changes performed every other day.

### Adipogenesis of pADMSCs

The isolated pADMSCs were expanded in tissue culture flasks and maintained in DMEM media supplemented with 10% v/v FBS and 1% v/v P/S. Upon confluency, the cells were differentiated into adipocytes using a similar technology devised by Zagury Y et al.^29^ but without the induction medium. The key to this differentiation is via the specific scaffold that is proprietary to the research group. The developed cells were cultured in DMEM media supplemented with chemical cues over 7 days. After that, the differentiated adipocytes were collected and characterised according to the adipocyte marker, LipidTOX™ Red neutral lipid stain, through qualitative and quantitative methods before studies were done.

### Co-culture of pADMSCs derived muscle and fat cells

On Day 17 of pADMSCs differentiation in the scaffold, 1 × 10^5^ pADMSC-derived adipocytes were seeded on top of the same scaffold following the same cell seeding strategy. The co-culture was maintained in a serum-reduced condition for 5 days, with media changes every other day.

### Cell viability

On PM days 2 and 7 and DM day 7, the scaffolds were washed with PBS and stained using live/dead staining kits (Life Technologies, Germany, L3224) containing calcein AM and ethidium homodimer-1 for labelling live and dead cells. A working solution of calcein AM (2 µM) and ethidium homodimer-1 (4 µM) in PBS, along with Hoechst 33342 (Thermofisher, H3570) for nuclei staining, was added to samples and incubated at 37 °C for 30 min. Excess dye solutions were removed through PBS washing. The stained cells were visualised using a Nikon A1RHD25 Confocal microscope at various time points, and the images were processed in NIS- Elements Imaging Software. The pixel dimensions were 4.29 µm/px (×4), 1.7 µm/px (×10) and 0.86 µm/px (×20). The image bit depth is 12 bits. Emission and excitation wavelengths for DAPI were 450 and 403.7 nm, for FITC were 525 and 488.6 nm, and for mCherry were 595 and 561.6 nm. Images taken post-Z stack were transformed into extended depth of focus (EDF) images and 3D view renderings. The PrestoBlue^TM^ assay (Thermofisher, A13262) was conducted on DPS with cells cultured on days 2, 4 and 7 and quantified according to the manufacturer’s instructions. Data were analysed using GraphPad Prism (software version 9.5.0). Statistical significance was determined through Brown–Forsythe and Welch ANOVA tests.

### Immunofluorescence of C2C12 differentiated muscle cells

The proliferated and differentiated C2C12 cells cultured on DPS were washed twice with PBS and fixed in 4% v/v paraformaldehyde (PFA, Boster Bio, AR1068) at room temperature for 15 min. To permeabilise the cells, 0.5% v/v Triton X-100 (Sigma, T8787) in PBS was applied for 10 min, followed by blocking with 5% v/v BSA (Sigma, A2153) in PBS for 1 h. Diluted primary antibodies, including Myosin Heavy Chain (MHC) (10 µg/mL, Abcam, #ab281901) and Myogenin (MYOG) (4 µg/mL, Abcam, #ab124800), were added to individual samples and incubated overnight at 4 °C. After three washes with washing buffer (PBS with 0.05% w/v BSA), the cells were incubated with secondary antibodies, goat anti-mouse IgG (H + L) Alexa Fluor 488 and 633 (1:1000, Thermofisher, A11008 and A21070) for 1 h. For F-actin staining samples, Alexa Fluor™ 488 Phalloidin (Thermofisher, A12379) was added and incubated for 30 min. Following additional washes, Hoechst 33342 was added for nuclei staining and incubated at room temperature for 30 min^27^. The samples were rewashed and imaged using a Nikon A1RHD25 confocal microscope at various time points and the images were processed in NIS- Elements Imaging Software. The pixel dimensions were 0.86 µm/px (×20). The image bit depth is 12 bits. Emission and excitation wavelengths for DAPI were 450 and 403.7 nm, for FITC were 525 and 488.6 nm, and for Cy5 were 700 and 640 nm. Images taken post-Z stack were transformed into extended depth of focus (EDF) images.

### Assessment of cell alignment on DPS

The assessment of cell alignment was conducted on Day 31 of C2C12 cell proliferation on DPS. To perform this analysis, the cells were fixed using 4% v/v paraformaldehyde and stained with Phalloidin 488 (Life Technologies, USA) for F-actin, as well as Hoechst 33342. Subsequently, the samples were subjected to imaging using a Nikon A1RHD25 confocal microscope at ×10 magnification. The alignment and orientation of the cells were determined using the OrientationJ plugin within ImageJ, which visualises cell alignment by assigning similar colours to cells with similar orientations. The orientation distribution was generated in the form of a histogram, representing the distribution of cell alignment angles. To quantify the average alignment, the Matlab CircStat toolbox was utilised, resulting in the calculation of a Kappa value within the range of 0-1.

### Immunofluorescence of pADMSCs differentiated muscle cells

The proliferated and differentiated pADMSCs cultured on DPSs were washed twice with PBS and fixed in 4% v/v PFA at room temperature for 15 min. To permeabilise the cells, 0.5% v/v Triton X-100 in PBS was applied for 10 min, followed by blocking with 5% w/v BSA in PBS for 1 h. Diluted primary antibodies, including Desmin (DES) (1:200, Sigma, #D1033) and Myosin Heavy Chain (MHC) (1:200, Sigma, #M8421) were added to individual samples and incubated overnight at 4 °C. After three washes with washing buffer (PBS with 0.05% w/v BSA), the cells were incubated with secondary antibodies, goat anti-mouse IgG (H + L) Alexa Fluor 488 and 633 (1:1000, Thermofisher, A32723TR and A-21050) for 1 h. For F-actin staining samples, Alexa Fluor™ 555 Phalloidin (Thermofisher, A34055) was added and incubated for 30 min. Following additional washes, Hoechst 33342 was added for nuclei staining and incubated at room temperature for 30 min^47^. The samples were washed 2–3 times and imaged using a Nikon A1RHD25 confocal microscope at various time points and the images were processed in NIS- Elements Imaging Software. The pixel dimensions were 0.86 µm/px (×20). The image bit depth is 12 bits. Emission and excitation wavelengths for DAPI were 450 nm and 403.7 nm, for FITC were 525 and 488.6 nm, for mCherry were 595 and 561.6 nm, and for Cy5 were 700 and 640 nm. Images taken post-Z stack were transformed into extended depth of focus (EDF) images.

### RNA extraction

Samples were collected at various time points during the differentiation period to analyse muscle gene and protein expression. RNA was extracted using the PureLink® RNA Mini Kit (Thermofisher, 12183018A) according to the manufacturer’s instructions. Briefly, scaffolds were washed with PBS and minced using sterile tweezers. Then, they were homogenised in a tube containing cell lysis buffer plus β-mercaptoethanol, after which they were centrifuged 12,000×*g* for 2 min before supernatants were extracted into a new 2-mL tube containing 70% v/v ethanol at a 1:1 ratio. Samples were then transferred into a column and centrifuged for 15 s at 12,000 × *g*. Wash buffer I was added into the column and then centrifuged for 15 s at 12,000×*g*. The spin cartridge was placed in a new collection tube, and wash buffer II was added to the column, which was 15 s at 12,000×*g*. This was repeated, and the columns were centrifuged for 15 s at 12,000×*g*. Dnase and Rnase- Free double distilled water (30 μL) was added to the column, incubated for 1 min, and centrifuged for 2 min at 12,000×*g*. The RNA concentration was measured using a NanoDrop (Thermo Fisher Scientific).

### Quantitative PCR (qPCR)

qPCR was performed in triplicate using the iTaq universal probes One-Step Kit (Bio-Rad, 1725141) with Taqman expression probes—Rn18s, Myosin Heavy Polypeptide 1 (MYH1) and MYOG (Thermofisher—Mm03928990_g1, Mm01332489_m1, and Mm00446194_m1) following the manufacturer’s instructions. 50 ng of RNA was used for each reaction, according to the manufacturer’s instructions. The reaction was run in the CFX96 Dx System (BIO-RAD, USA). Data were analysed using GraphPad Prism (software version 9.5.0). Statistical significance was determined through one-way ANOVA with Tukey’s multiple comparisons tests. The results are presented as 2−Δct and normalised to the Rn18S housekeeping gene.

### Creatinine kinase (CK) assay

The scaffolds with cells at different time points were lysed using M-PER Mammalian Protein Extraction Reagent (78501, Thermo Scientific) supplemented with protease inhibitor and phosphatase inhibitor cocktail (1:100) (Thermofisher, 78446). The lysed cell solution was centrifuged (4 °C, 14,000×*g*, 10 min), and the extracted protein supernatant was stored at −20 °C for further measurement of total protein and CK quantification. CK activity was calculated corresponding to the manufacturer’s procedure (MAK116, Sigma Aldrich). Briefly, 10 µL of protein extract was added to a 96-well plate, and 100 mL of reconstituted reagent and incubated at 37 °C. The incubated samples on 96-well plate was measured at 340 nm at 20 and 40 min using a microplate reader (TECAN, infinite M200pro). The final CK activity was normalised by total protein content, which was determined by BCA protein assay (23225, Thermo Scientific). CK activity was calculated using the absorbance of 20 and 40 min per the protocol-instructed formula, as shown in Eq. (1) below^27^. Data were analysed using GraphPad Prism (software version 9.5.0). Statistical significance was determined through one-way ANOVA with Tukey’s multiple comparisons tests.

1 $${\rm{CK}}\; {\rm{activity}}=\frac{\left(A340\right)40{\rm{min}} -{\left(A340\right)}_{20{\rm{min}} }}{{\left(A340\right)}_{{{\rm {Cal}}}}-{\left(A340\right)}_{{{\rm {Blank}}}}}\times 150$$

### Field emission scanning electron microscope (FESEM) imaging

The DPS with differentiated muscle cells were washed with PBS and fixed with 4% v/v PFA solution for 30 min. Fixed cells were treated with ethanol gradient concentration (30–100%) intended for dehydration and followingly dried the DPS with critical point drying (CPD) (Leica EM CPD300 Critical Point Dryer). Dried DPS were further coated with platinum (Pt) to visualise under SEM. JSC-1200 fine coater (JEOL, Japan) was used to sputter coat DPS for a thickness of 15 nm. Sputter-coated DPS were then analysed using FESEM (JSM-6701F, JEOL, USA). Morphological analysis of pADMSCs and C2C12 grown on the DPS were imaged using FESEM.

### Immunofluorescence and LipidTOX staining

At Day 21, DPS co-cultured with pADMSC differentiated muscle and fat cells were washed with PBS and fixed in 4% v/v PFA at room temperature for 15 min. To permeabilise the cells, 0.1% v/v Saponin (Sigma, 47036) in a blocking buffer (5% w/v BSA) was applied for 1 h. Diluted primary antibodies, including Myosin Heavy Chain (MHC) (1:200, Sigma, #M8421) and Desmin (DES) (1:200, Sigma, #D1033), were added to individual samples and incubated overnight at 4 °C. After three washes with washing buffer (PBS with 0.05% BSA & 0.1% v/v Saponin), the cells were incubated with secondary antibodies, goat anti-mouse IgG (H + L) Alexa Fluor 633 and 488 (1:1000, Thermofisher, A-21050 and A32723TR) for 1 h. For F-actin staining samples, Alexa Fluor™ 555 Phalloidin (Thermofisher, A34055) was added and incubated for 30 min. Following additional washes, Hoechst 33342 was added for nuclei staining, incubated at room temperature for 30 min, and washed with washing buffer three times. LipidTox reagent (Thermofisher, H34475) was added to the DPS in a 1:200 ratio in PBS and incubated for 30 min at room temperature. The samples were washed again and imaged using a Nikon A1RHD25 confocal microscope and the images were processed in NIS- Elements Imaging Software. The pixel dimensions were 0.86 µm/px (×20) and 0.43 µm/px (×40). The image bit depth is 12 bits. Emission and excitation wavelengths for DAPI were 450 and 403.7 nm, for FITC were 525 and 488.6 nm, for mCherry were 595 and 561.6 nm, and for Cy5 were 700 and 640 nm. Images taken post-Z stack were transformed into extended depth of focus (EDF) images.

### Cooking

All samples [DPS with cells (CM prototype), DPS, pork loin] used for cooking were cut to the same dimension and subjected to the relevant treatment as described above. Based on an adaptation of a method by Ben-Arye et al.^43^, the samples were individually pan-fried in olive oil (Bertolli, Spain) on both sides for 30 s each (using the ‘frying’ mode of the induction cooker (PPIC887, PowerPac, Singapore) at 80 °C, 600 W) and placed on a baking paper to remove any excess oil. All samples were cooled to room temperature before TPA was performed.

### Texture profile analysis (TPA) of CM prototype

TPA was performed with a TA.XTPlusC Texture Analyser (Stable Micro Systems, UK) using a method adapted by Godschalk-Broers et al.^30^ to determine the hardness, springiness, cohesiveness and chewiness. A 75 mm compression platen (P/75) with a 1 kg load cell was used for this study. Despite efforts to maintain a consistent sample size, there were slight variations in thickness and length. The cooked CM prototype, raw scaffolds, and pork loin were then subjected to a double compression test with the following conditions: 50% deformation, 5 s between the two compression cycles, compression speed of 2 mm/s, and data acquisition rate of 500 pulses per second (PPS). The textural parameters were then calculated using the Exponent Connect software (Exponent Connect, Stable Microsystems, UK). These measurements were performed with five replicates.

### Statistical analysis

The statistical analysis was conducted using a computerised statistical programme, GraphPad Prism software (version 9.5.0). To analyse the cell viability data from the PrestoBlue^TM^ assay, the data were analysed, and the statistical significance was determined using the Brown–Forsythe and Welch ANOVA tests. Quantitative PCR (qPCR) analysis of muscle-related genes, CK activity, and TPA data was analysed, and statistical significance was determined through one-way ANOVA with Tukey’s multiple comparisons tests. Error bars represent the standard deviation of the mean.

### Reporting summary

Further information on research design is available in the Nature Research Reporting Summary linked to this article.

### Supplementary information

Revised Supplementary Main File

Supplementary - PCR Data

Reporting summary

S1

S2

S3

S4

## Data Availability

The authors declare that all data supporting the findings of this study are available within the paper and its supplementary information files. Further data, including source data supporting the findings of this study, are available from the corresponding author upon request.
