# Supplementary material for: Decellularised plant scaffolds facilitate porcine skeletal muscle tissue engineering for cultivated meat biomanufacturing
Source: NPJ Sci Food. 2024 May 3;8:25. doi: 10.1038/s41538-024-00262-1 (PMC11068908; doi:10.1038/s41538-024-00262-1)
Supplement: Supplementary file 1 — Revised Supplementary Main File [file 41538_2024_262_MOESM1_ESM.pdf]

Supplementary Information

Decellularised Plant Scaffolds Facilitate Porcine Skeletal Muscle Tissue Engineering for Cultivated Meat Biomanufacturing

Priyatharshini Murugan[1], Wee Swan Yap[1], Hariharan Ezhilarasu[1], Ratima Suntornnond[1], Quang Bach Le[1], Satnam Singh[1], Jasmine Si Han Seah[2], Pei Leng Tan[2], Zhou Weibiao[3], Lay Poh Tan[2], Deepak Choudhury[1]*

[1]Biomanufacturing Technology, Bioprocessing Technology Institute (BTI),

*Agency for Science, Technology, and Research (A*STAR)*

[2]School of Materials Science and Engineering, Nanyang Technological University, Singapore

[3] Department of Food Science and Technology, National University of Singapore, Singapore

**Correspondence: Deepak_Choudhury@bti.a-star.edu.sg*

Supplementary Figures

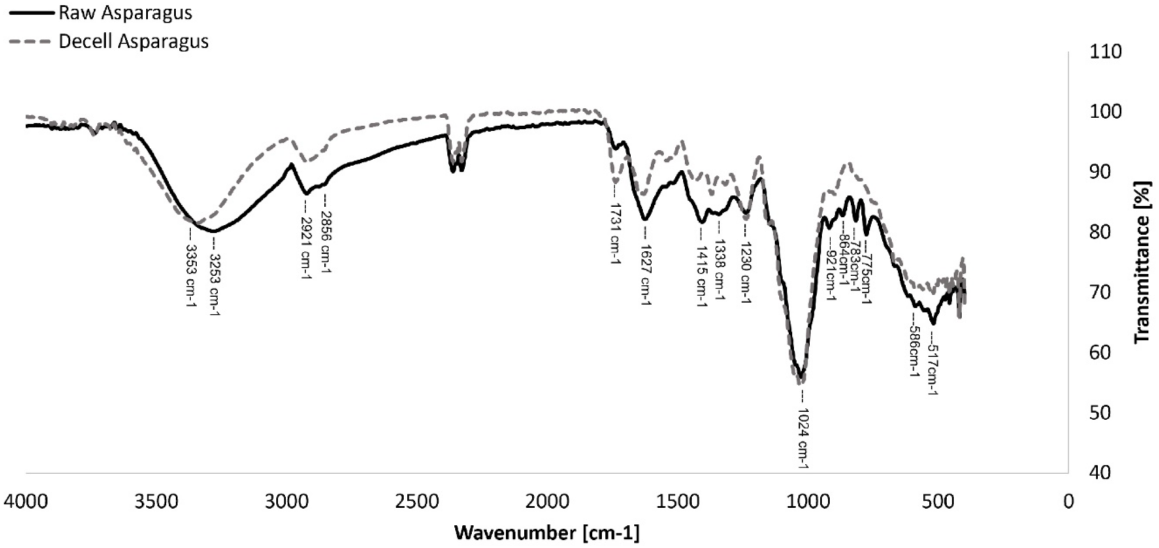

Supplementary Figure 1. The FTIR spectrum for the raw and decellularised asparagus scaffold. A band at 3253 cm^{-1} indicates the presence of hydroxyl groups in polysaccharides, possibly related to hydrogen bond vibrations in cellulose. The region between 3800-3000 cm^{-1} links to the crystalline structure of cellulose. A band at 2923 cm^{-1} and 2910 cm^{-1} represents characteristic asymmetric stretching vibrations of the CH_2 group in cellulose. Narrow bands at 1730-1740 cm^{-1} suggest stretching of C=O and C-O bonds in hemicellulose. A peak at 1627 cm^{-1} is reported to be stretching the C=C and C=O lignin aromatic ring and the band between 1410-1435 cm^{-1} is the deformation of lignin CH2 and CH3. The range between 1200 cm^{-1} and 1000 cm^{-1} is the region of hemicellulose, cellulose, and pectin, which attained a maximum value of around 1020 cm^{-1} due to C-O stretching. The peak at 897 cm^{-1} indicates the β-glycosidic linkage between the monomers of cellulose. All the above-mentioned bands and peaks were reported in both raw and decellularised samples, showing that even after the decellularisation, major plant macromolecules were kept preserved.

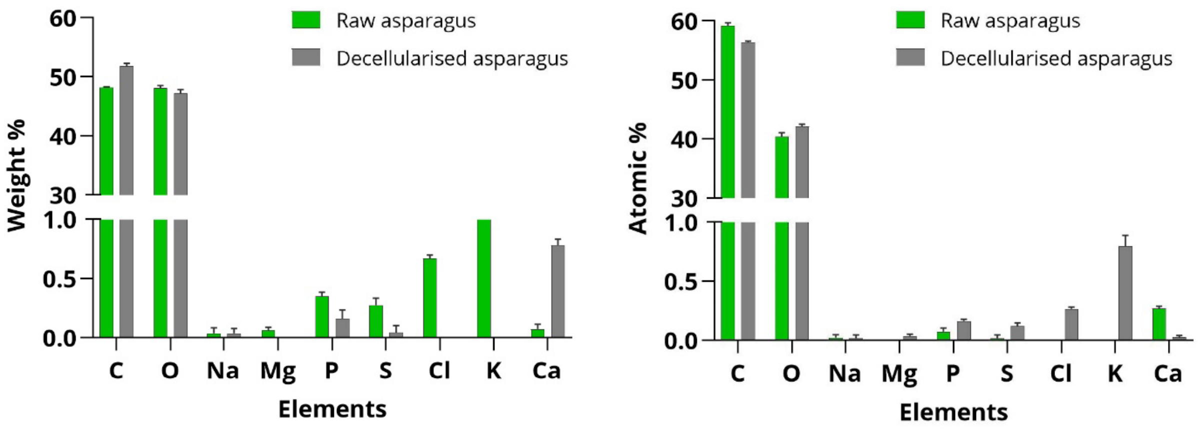

Supplementary Figure 2. Energy Dispersive X-ray **(**EDX) elemental analysis of raw and decellularised asparagus scaffolds.

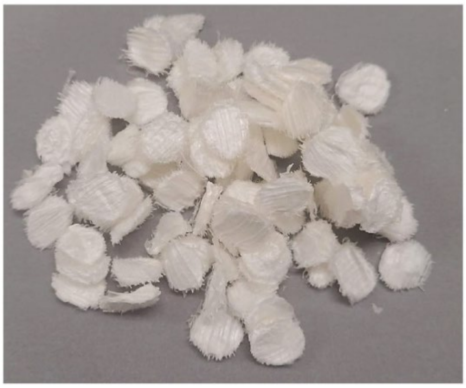

Supplementary Figure 3. Visual image of 100 decellularised asparagus scaffolds (freeze-dried).

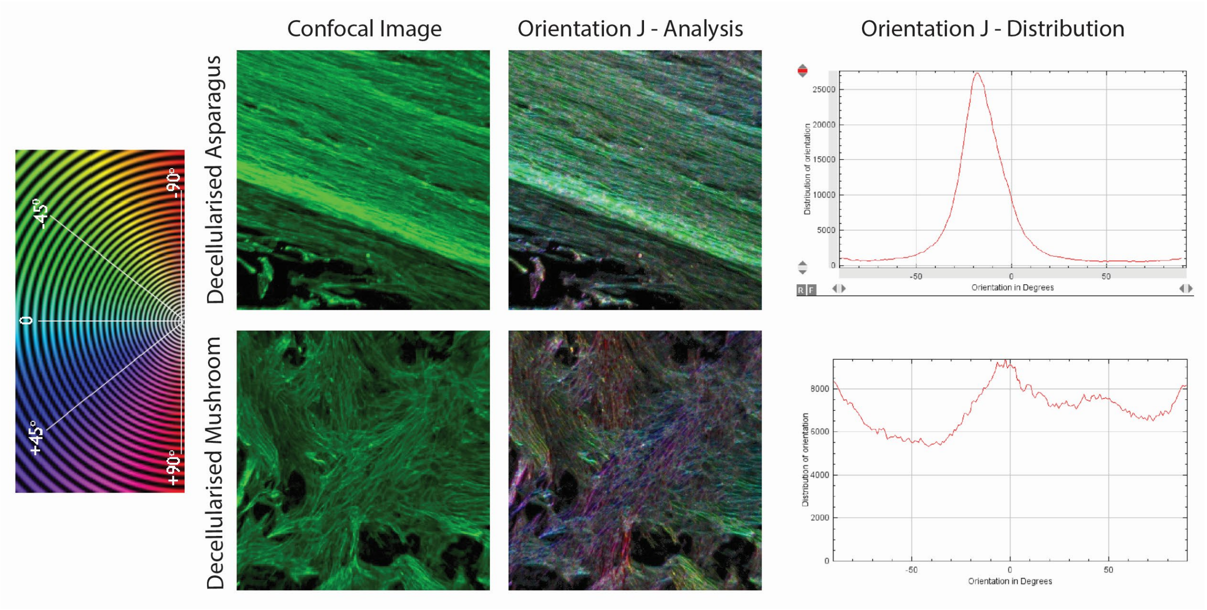

Supplementary Figure 4. The alignment of C2C12 cells on decellularised asparagus and mushroom substrates was examined. A directional analysis colour survey was conducted to assess the alignment of C2C12 cell proliferation on these scaffolds on Day 31. Each distinct colour within the survey corresponds to a different alignment angle, aiding in visualising and distinguishing the direction of cell alignment in these unique environments. The vascular bundle arrangement of the asparagus scaffold promotes cell alignment, in contrast to decellularised mushroom scaffolds, which exhibit undefined cell alignment. Because of the alignment features of the scaffold, asparagus was selected in this study.

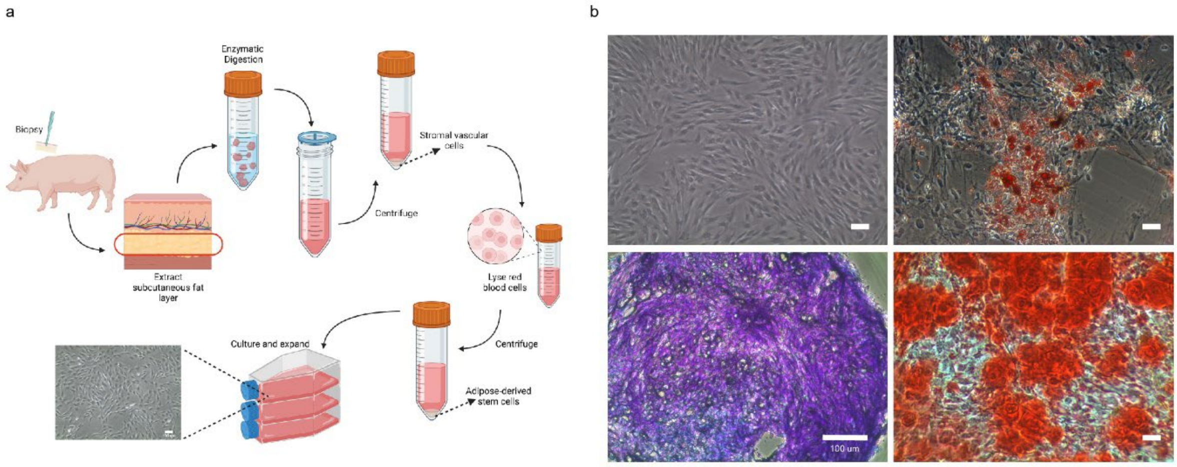

Supplementary Figure 5. pADMSCs isolation process and characterisation **a**. Isolation of pADMSCs from porcine adipose tissue; **b**, pADMSCs on tissue culture plate (TCP), cultured with growth media (top, left), pADMSCs stained positive with Oil Red O after 21 days of culture in adipogenic media (top, right), pADMSCs stained positive with Toluidine Blue O after 21 days of culture in chondrogenic media (bottom, left), pADMSCs stained positive with Alazarin Red S after 21 days of culture in osteogenic media (bottom, right), scale bar, 100 µm.

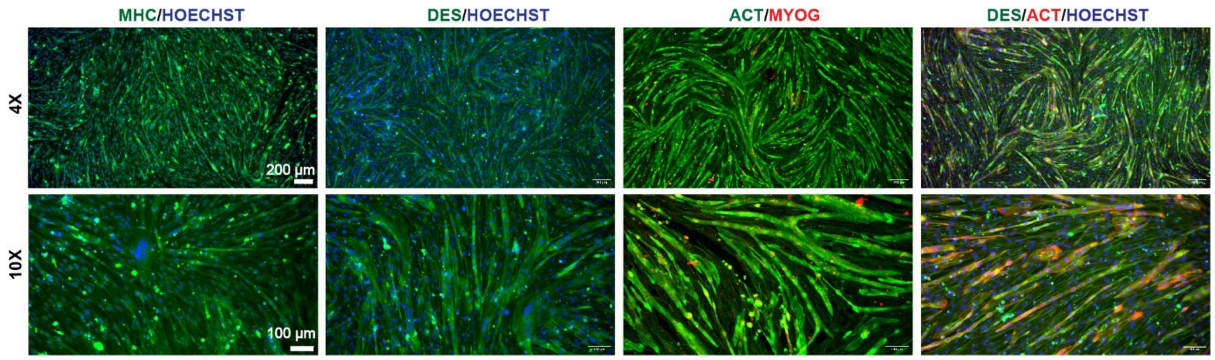

Supplementary Figure 6. Representative immunofluorescence images of pADMSC differentiated into muscle cells in 2D tissue culture plate using differentiation media (DM). Actin, ACT; Myosin Heavy Chain, MHC; Myogenin, MYOG; Hoechst. Scale bar, 100 µm (10X magnification); 200 µm (4X magnification).

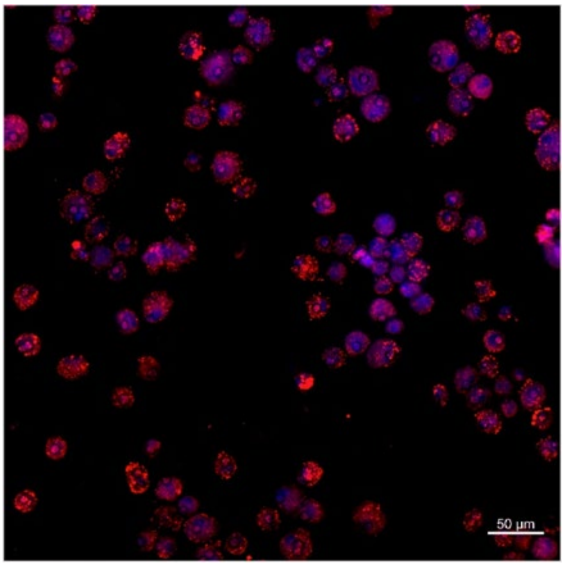

Supplementary Figure 7. Representative image of lipid droplets stained with LipidTOX Red neutral lipid stain (red fluorescence) and nuclei stained with Hoechst (blue fluorescence) in differentiated adipocytes after 21 days of differentiation. Scale bar = 50 µm.

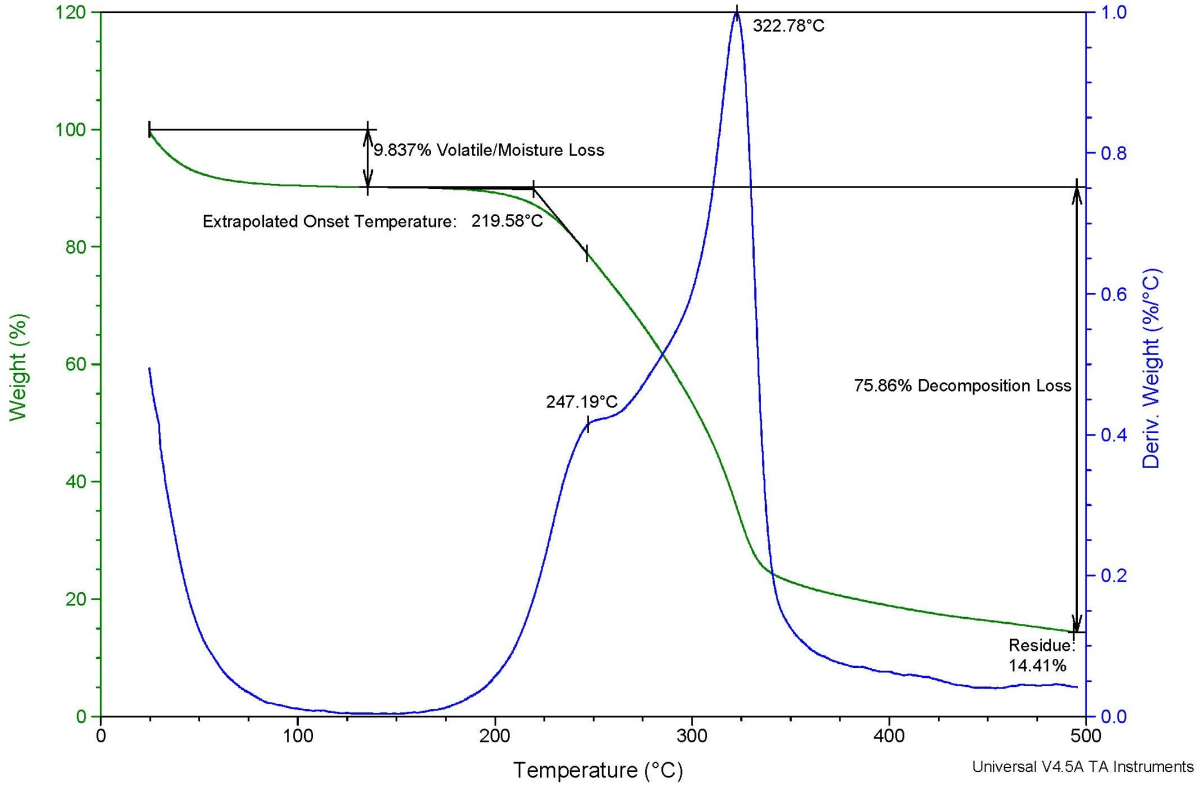

Supplementary Figure 8. Thermogravimetric analysis (TGA) of decellularised asparagus scaffold. The graph depicted the percentage weight change of the scaffold when the temperature was increased from 25 to 500°C at a rate of 10°C /min. The samples are stable at physiological temperatures, and the first decomposition observed on the graphs between 25 to 100°C was due to weight loss by the removal of moisture and other volatiles from the scaffold. It can also be observed that the scaffolds are stable up to 200°C. The scaffold showed different onset decomposition temperatures at 219°C. The greatest rate of weight change was also experienced at 247.19 °C and 332.78 °C, respectively.

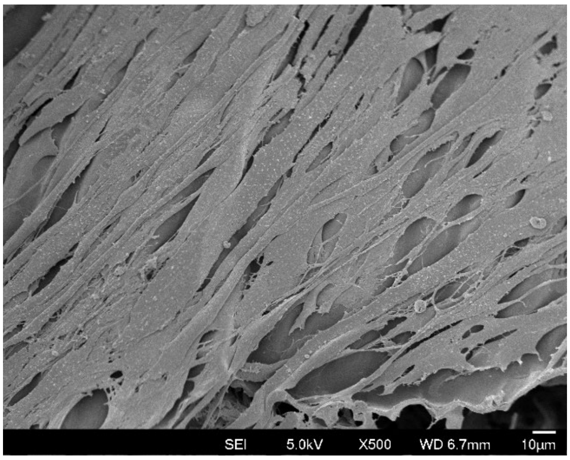

Supplementary Figure 9. Raw images of the Fig. 2c - SEM image of C2C12 differentiated muscle cells (Day 9) on scaffolds.

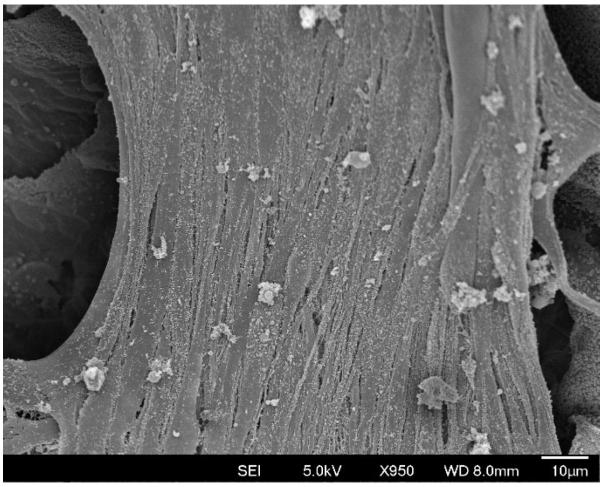

Supplementary Figure 10. Raw images of the Fig. 3e - SEM image of pADMSC differentiated muscle cells (Day 17) on scaffolds.

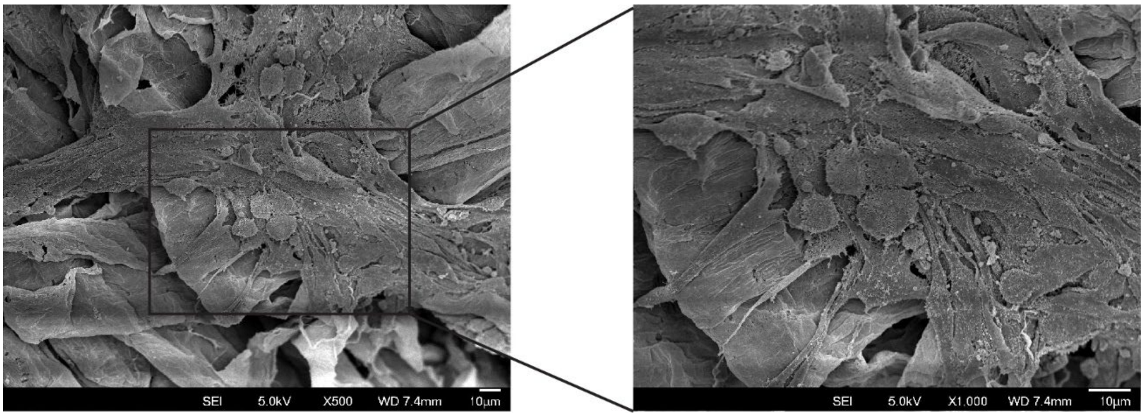

Supplementary Figure 11. Raw images of the Fig. 4b - SEM of pADMSCs differentiated muscle cells, (Day 22) and fat (Day 5) on scaffolds.

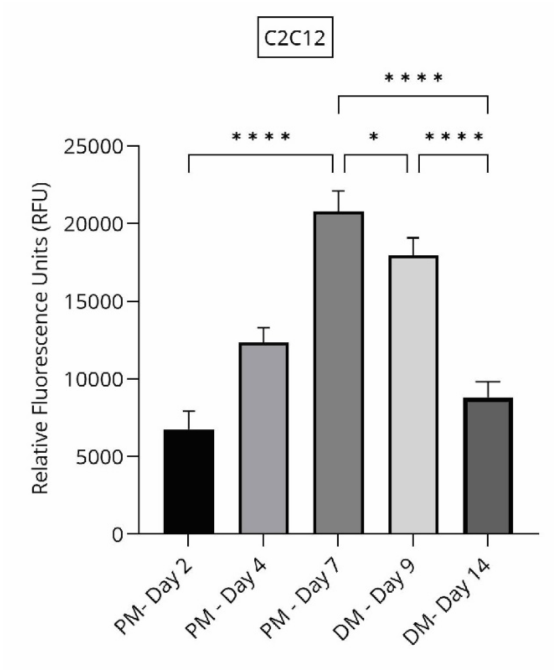

Supplementary Figure 12. Prestoblue cell viability assay of C2C12 cells in decellularised asparagus scaffold. Upon achieving confluency on Day 7, the transition from proliferation media (PM) to differentiation media (DM) (DMEM with reduced serum) was performed to induce cell fusion and myotube formation. On DM-Day 9, cell death was notably lower compared to DM-Day 14, where over-confluent matured cells exhibited signs of cell death or detachment from the scaffold. To optimize the experimental protocol, the study was intentionally concluded at DM-Day 9.

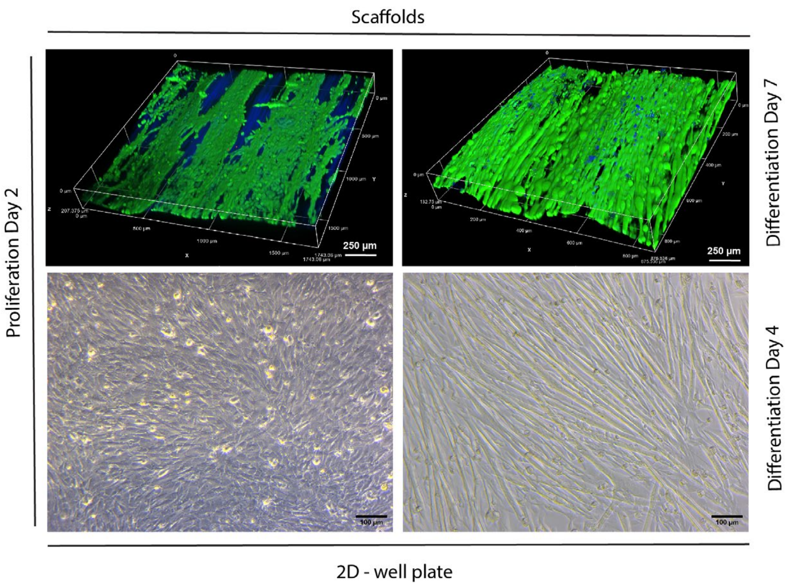

Supplementary Figure 13. The proliferation of C2C12 cells on decellularized asparagus scaffolds and 2D cell culture plates on Day 2. Additionally, it depicts the differentiation of C2C12 cells on scaffolds by Day 7 and on 2D cell culture plates by Day 4. While cells in 2D culture plates exhibit rapid proliferation, reaching confluency by Day 2, the decellularized asparagus scaffolds display a distinct proliferation pattern, achieving confluency on Day 7. The 3D structure of the scaffolds reveals a 2.5D and partially 3D configuration, with cells adhering to the fibrous architecture and attaching to major and minor grooves. This unique growth behaviour introduces variations compared to the swift confluency observed in 2D cultures. Due to these differences in cellular growth behaviour and proliferation patterns, employing a 2D tissue culture plate as a positive control for assessing cell viability on the scaffold is deemed unsuitable.

Supplementary Videos

Supplementary Video 1: C2C12 myoblast differentiated into myotubes in decellularised plant scaffold immuno-stained with MHC (green) antibody and Hoechst (blue).

Supplementary Video 2: Co-culture of pADMSC differentiated muscle and fat cells in decellularised plant scaffold immune-stained with actin (red), LipidTox (green) and Hoechst (blue)

Supplementary Video 3: Co-culture of pADMSC differentiated muscle and fat cells in decellularised plant scaffold immune-stained with desmin (magenta), LipidTOX (green) and Hoechst (blue)

Supplementary Video 4: Pan-frying cooking video of CM analogue
