# Supplementary material for: Decellularised plant scaffolds facilitate porcine skeletal muscle tissue engineering for cultivated meat biomanufacturing
Source: NPJ Sci Food. 2024 May 3;8:25. doi: 10.1038/s41538-024-00262-1 (PMC11068908; doi:10.1038/s41538-024-00262-1)
Supplement: Supplementary file 3 — Reporting summary [file 41538_2024_262_MOESM3_ESM.pdf]

nature portfolio

Corresponding author(s): Deepak Choudhury (NPJSCIFOOD-01312R)

Last updated by author(s): Feb 27, 2024

Reporting Summary

Nature Portfolio wishes to improve the reproducibility of the work that we publish. This form provides structure for consistency and transparency in reporting. For further information on Nature Portfolio policies, see our Editorial Policies and the Editorial Policy Checklist.

Statistics

For all statistical analyses, confirm that the following items are present in the figure legend, table legend, main text, or Methods section.

n/a	Confirmed	
☐	☒	The exact sample size (*n*) for each experimental group/condition, given as a discrete number and unit of measurement
☒	☐	A statement on whether measurements were taken from distinct samples or whether the same sample was measured repeatedly
☐	☒	The statistical test(s) used AND whether they are one- or two-sided<br>*Only common tests should be described solely by name; describe more complex techniques in the Methods section.*
☒	☐	A description of all covariates tested
☒	☐	A description of any assumptions or corrections, such as tests of normality and adjustment for multiple comparisons
☐	☒	A full description of the statistical parameters including central tendency (e.g. means) or other basic estimates (e.g. regression coefficient) AND variation (e.g. standard deviation) or associated estimates of uncertainty (e.g. confidence intervals)
☒	☐	For null hypothesis testing, the test statistic (e.g. F, t, r) with confidence intervals, effect sizes, degrees of freedom and P value noted<br>*Give P values as exact values whenever suitable.*
☒	☐	For Bayesian analysis, information on the choice of priors and Markov chain Monte Carlo settings
☒	☐	For hierarchical and complex designs, identification of the appropriate level for tests and full reporting of outcomes
☒	☐	Estimates of effect sizes (e.g. Cohen's d, Pearson's r), indicating how they were calculated

Our web collection on statistics for biologists contains articles on many of the points above.

Software and code

Policy information about availability of computer code

Data collection	n/a
Data analysis	1. The volume, surface area of the pores and porosity of the decellularised scaffoldwere quantified and analysed using the CTAn software (Bruker Industry, USA). 2. Confocal Images were processed using in NIS- Elements Imaging Software. 3. . The alignment and orientation of the cells were determined using the OrientationJ plugin within ImageJ. 4. qPCR data were analysed using GraphPad Prism (software version 9.5.0). Statistical significance was determined through one-way ANOVA with Tukey's multiple comparisons tests. 5. The textural parameters were then calculated using the Exponent Connect software (Exponent Connect, Stable Microsystems, United Kingdom). 6. The statistical analysis was conducted using a computerised statistical program. To analyse the cell viability data from the Presto-blue assay, the data were analysed using GraphPad Prism software (version 9.5.0). Statistical significance was determined using the Brown-Forsythe and Welch ANOVA tests.

For manuscripts utilizing custom algorithms or software that are central to the research but not yet described in published literature, software must be made available to editors and reviewers. We strongly encourage code deposition in a community repository (e.g. GitHub). See the Nature Portfolio guidelines for submitting code & software for further information.

Data

Policy information about availability of data

All manuscripts must include a data availability statement. This statement should provide the following information, where applicable:

- Accession codes, unique identifiers, or web links for publicly available datasets
- A description of any restrictions on data availability
- For clinical datasets or third party data, please ensure that the statement adheres to our policy

The authors declare that all data supporting the findings of this study are available within the paper and its supplementary information files. Further data, including source data supporting the findings of this study, are available from the corresponding author upon request.

Research involving human participants, their data, or biological material

Policy information about studies with human participants or human data. See also policy information about sex, gender (identity/presentation), and sexual orientation and race, ethnicity and racism.

Reporting on sex and gender	n/a
Reporting on race, ethnicity, or other socially relevant groupings	n/a
Population characteristics	n/a
Recruitment	n/a
Ethics oversight	n/a

Note that full information on the approval of the study protocol must also be provided in the manuscript.

Field-specific reporting

Please select the one below that is the best fit for your research. If you are not sure, read the appropriate sections before making your selection.

☒ Life sciences ☐ Behavioural & social sciences ☐ Ecological, evolutionary & environmental sciences

For a reference copy of the document with all sections, see nature.com/documents/nr-reporting-summary-flat.pdf

Life sciences study design

All studies must disclose on these points even when the disclosure is negative.

Sample size	The selection of a sample size for quantitative measurements, including Young's modulus, Prestoblue assay, and texture profile analysis, was set at n=5. For confocal imaging (immunofluorescence, live-dead analysis), SEM imaging, and CK analysis, a sample size of n=3 was chosen to ensure reliable, reproducible, and valid results.
Data exclusions	n/a
Replication	We confirm that the experimental findings were successfully reproducible in most attempts, except under conditions where cell culture contaminations occurred.
Randomization	n/a
Blinding	n/a

Reporting for specific materials, systems and methods

We require information from authors about some types of materials, experimental systems and methods used in many studies. Here, indicate whether each material, system or method listed is relevant to your study. If you are not sure if a list item applies to your research, read the appropriate section before selecting a response.

Materials & experimental systems

n/a	Involved in the study	
☐	☒	Antibodies
☐	☒	Eukaryotic cell lines
☒	☐	Palaeontology and archaeology
☒	☐	Animals and other organisms
☒	☐	Clinical data
☒	☐	Dual use research of concern
☐	☒	Plants

Methods

n/a	Involved in the study	
☒	☐	ChIP-seq
☒	☐	Flow cytometry
☒	☐	MRI-based neuroimaging

Antibodies

Antibodies used

1. C2C12 mouse muscle differentiation - Immunofluorescence (IF) staining. Primary antibodies: Myosin Heavy Chain (MHC) (10 µg/mL, Abcam, #ab281901) and Myogenin (MYOG) (4 µg/mL, Abcam, #ab124800). Secondary antibodies: goat anti-mouse IgG 419 (H+L) Alexa Fluor 633 and 488 (1:1000, Thermofisher, A21070 and A11008). 2 Porcine adipose derived mesenchymal stem cell muscle differentiation - IF staining. Primary antibodies: Myosin 430 Heavy Chain (MHC) (1:200, Sigma, #M8421) and Desmin (DES) (1:200, Sigma, #D1033). Secondary antibodies: goat anti-mouse IgG (H+L) Alexa Fluor 633 and 488 (1:1000, Thermofisher, A-434 21050 and A32723TR).

Validation

Data provided in the manuscript.

Eukaryotic cell lines

Policy information about cell lines and Sex and Gender in Research

Cell line source(s)

1. C2C12 mouse cell line, obtained from the American Type Culture Collection (ATCC CRL – 327 1772™), was employed in this study. 2. Mesenchymal stem cells (pADMSC) derived from porcine adipose tissue were extracted and collected from the subcutaneous adipose tissues of the pigs.

Authentication

1. C2C12 authentication done by the company provider ATCC. 2. pADMSC authentication done by multipotency testing of adipogenesis, chondrogenesis and osteogenesis (mentioned in the supplementary information)

Mycoplasma contamination

1. C2C12 mycoplasma contamination was tested by the company provider ATCC. 2. pADMSC mycoplasma contamination was not tested.

Commonly misidentified lines
(See ICLAC register)

N/a

Dual use research of concern

Policy information about dual use research of concern

Hazards

Could the accidental, deliberate or reckless misuse of agents or technologies generated in the work, or the application of information presented in the manuscript, pose a threat to:

No	Yes	
☒	☐	Public health
☒	☐	National security
☒	☐	Crops and/or livestock
☒	☐	Ecosystems
☒	☐	Any other significant area

Experiments of concern

Does the work involve any of these experiments of concern:

No	Yes	
☒	☐	Demonstrate how to render a vaccine ineffective
☒	☐	Confer resistance to therapeutically useful antibiotics or antiviral agents
☒	☐	Enhance the virulence of a pathogen or render a nonpathogen virulent
☒	☐	Increase transmissibility of a pathogen
☒	☐	Alter the host range of a pathogen
☒	☐	Enable evasion of diagnostic/detection modalities
☒	☐	Enable the weaponization of a biological agent or toxin
☒	☐	Any other potentially harmful combination of experiments and agents

Plants

Seed stocks	White asparagus (Asparagus officinalis) for the preparation of a decellularised plant scaffold was obtained from a local grocery store.
Novel plant genotypes	n/a
Authentication	n/a
